# Normal visuospatial function in unilateral vestibulopathy: on the challenge of group differences within normal reference data

**DOI:** 10.3389/fneur.2023.1334277

**Published:** 2023-12-13

**Authors:** Christoph Helmchen, Anja Fellbrich, Andreas Sprenger

**Affiliations:** ^1^Department of Neurology, University Hospital Schleswig-Holstein, Lübeck, Germany; ^2^Center of Brain, Behavior and Metabolism (CBBM), University of Lübeck, Lübeck, Germany; ^3^Institute of Psychology II, University Lübeck, Lübeck, Germany

**Keywords:** visuospatial, vestibulopathy, normal reference range values, cognition, memory

We read with great interest the article by Oh and coworkers about “*Visuospatial cognition in acute unilateral peripheral vestibulopathy*” in the September edition of this journal Frontiers in Neurology (Section Neuro-Otology). The authors tested visuospatial perception by the Visual Object and Space Perception (VOSP) battery established by Warrington and James (1), validated by Rapport et al. (2), and visuospatial memory by the Block design test (3) and the Corsi block-tapping test (4) within the first 2 days (acute phase) and 4 weeks (recovery phase) after symptom onset of acute unilateral vestibulopathy (UVF).

There are several animal studies showing that unilateral experimental vestibular lesions elicit signs of spatial memory and visuospatial deficits which have been related to vestibulo-cortical projections, in particular to the hippocampal formation (5–7). However, these findings have not convincingly been confirmed in patient studies yet (8–12) but there is some-though not consistent (13–15)-evidence that patients with bilateral vestibular failure reveal visuospatial deficits or even path navigation impairments in addition to volumetric changes of the hippocampus (8, 16, 17).

Based on this background, the study by Oh et al. investigated visuospatial perception and memory in a large cohort (*n* = 72) of patients with UVF. As a main result, the patients showed normal visuospatial functions in the acute and recovery phase of the disease according to the normal reference values established by the standardization sample of the test founders. It is important to realize that the statistical differences between healthy subjects and patients were found *within* normal limits. This needs to be kept in mind when the authors speculate on the potential origin of visuospatial perception and spatial memory impairments.

We felt encouraged to point out the readers's risk of misunderstanding these results since at least two related studies also interpreted values within the normal reference range to be pathological:

First, Ayar et al. (12) used the Judgement of Line Orientation test (JLO) (18) to test visuospatial function in 21 patients with acute UVF (mean disease duration: 15.6 days). Patients and healthy control subjects revealed normal values according to the normal reference range. Patients had significantly lower but normal values (21 to 26, normal: >19) and yet their data were considered as a pathological sign of visuospatial function, even despite the fact that the significant difference disappeared when depression and anxiety scores were taken into account (multiple regression analysis).

Second, very recently, Obermann et al. (19) compared cognitive functions in 65 patients with UVF and healthy subjects, including the JLO test (18). Patients had lower (23.7 vs. 26.4) but normal values (>19) in the JLO test 5 months after symptom onset, i.e., at a time when the visuospatial group differences in the study by Oh et al. had already resolved.

UVF usually persists over a few months which unfortunately was not tested in the follow-up examination by Oh et al. (20). This could have helped to elucidate if the group differences are related to the vestibular system, in particular since JLO values decreased with greater semicircular canal paresis in the study by Ayar et al. (12).

In all three studies, the significant group differences were interpreted as signs of visuospatial impairment. Since they refer to the normal reference range of the original test studies (1, 18) they lack convincing evidence that unilateral vestibulopathy elicit visuospatial perception and memory impairments. Based on the large cohort sizes of these UVF studies it appears rather unlikely that these particular cognitive functions are altered in UVF patients.

These analytic approaches raise the question whether statistical differences within the numeric range defined as being “normal” or “healthy” in the studies establishing these neuropsychological tests, should be used for a pathological interpretation, in particular when the differences cannot be confirmed by a follow-up examination of the same tests (20).

Investigations on subthreshold or premanifesting disorders have been found to be a valuable approach to distinguish trait and state signs in several neurogenetic and neurodegenerative disorders (21, 22) but group differences within normal limits are usually classified as state signs only when they turn pathological during follow-up investigations. In contrast, the group differences in the study by Oh et al. resolved after 4 weeks. We recommend follow-up studies on vestibular and visuospatial function to investigate whether the group differences within the normal reference range have any pathological, e.g. neurodegenerative meaning. We consider this an important clarification to reduce the readers' risk of misunderstanding and potential inappropriate citations of these important data.

## Author contributions

CH: Writing – original draft. AF: Formal analysis, Methodology, Validation, Writing – review & editing. AS: Methodology, Validation, Writing – review & editing.
